# Myocardial fibrosis in patients with myotonic dystrophy type 1: a cardiovascular magnetic resonance study

**DOI:** 10.1186/s12968-014-0059-z

**Published:** 2014-08-01

**Authors:** Helle Petri, Kiril Aleksov Ahtarovski, Niels Vejlstrup, John Vissing, Nanna Witting, Lars Køber, Henning Bundgaard

**Affiliations:** 1 Department of Cardiology, Copenhagen University Hospital, Rigshospitalet, Blegdamsvej 9, Copenhagen, 2100, Denmark; 2 Neuromuscular Research Unit, Department of Neurology, Copenhagen University Hospital, Rigshospitalet, Copenhagen, Denmark; 3 Unit for Inherited Cardiac Diseases, Copenhagen University Hospital, Rigshospitalet, Copenhagen, Denmark

**Keywords:** Myotonic dystrophy, Cardiovascular magnetic resonance, Myocardial fibrosis, Risk stratification, Sudden cardiac death

## Abstract

**Background:**

Myotonic dystrophy type 1 (DM1) is associated with increased cardiac morbidity and mortality. Therefore, assessment of cardiac involvement and risk stratification for sudden cardiac death is crucial. Nevertheless, optimal screening-procedures are not clearly defined. ECG, echocardiography and Holter-monitoring are useful but insufficient. Cardiovascular magnetic resonance (CMR) can provide additional information of which myocardial fibrosis may be relevant.

The purpose of this study was to describe the prevalence of myocardial fibrosis in patients with DM1 assessed by CMR, and the association between myocardial fibrosis and abnormal findings on ECG, Holter-monitoring and echocardiography.

**Methods:**

We selected 30 unrelated patients with DM1: 18 patients (10 men, mean age 51 years) with, and 12 patients (7 men, mean age 41 years) without abnormal findings on ECG and Holter-monitoring.

Patients were evaluated with medical history, physical examination, ECG, Holter-monitoring, echocardiography and CMR.

**Results:**

Myocardial fibrosis was found in 12/30 (40%, 9 men). The presence of myocardial fibrosis was associated with the following CMR-parameters: increased left ventricular mass (median (range) 55 g/m^2^ (43–83) vs. 46 g/m^2^ (36–64), p = 0.02), increased left atrial volume (median (range) 52 ml/m^2^ (36–87) vs. 46 ml/m^2^ (35–69), p = 0.04) and a trend toward lower LVEF (median (range) 63% (38–71) vs. 66% (60–80), p = 0.06). Overall, we found no association between the presence of myocardial fibrosis and abnormal findings on: ECG (p = 0.71), Holter-monitoring (p = 0.27) or echocardiographic measurements of left ventricular volumes, ejection fraction or global longitudinal strain (p = 0.18).

**Conclusion:**

Patients with DM1 had a high prevalence of myocardial fibrosis which was not predicted by ECG, Holter-monitoring or echocardiography. CMR add additional information to current standard cardiac assessment and may prove to be a clinically valuable tool for risk stratification in DM1.

## Background

Myotonic dystrophy type 1 (DM1) is an autosomal dominantly inherited neuromuscular disorder caused by an unstable expansion of a tri-nucleotide (CTG) repeat on chromosome 19 in the 3’ untranslated region of the myotonic dystrophy protein kinase gene [1].

The cardiac phenotype of DM1 is complex and includes an increased risk of conduction disturbances, arrhythmias, compromised systolic and diastolic function and an approximately three-fold higher risk of sudden cardiac death (SCD) compared to age-matched healthy controls [2]-[4]. Additionally, disease progression is unpredictable, necessitating regular and repeated cardiac assessment and risk stratification for SCD [3],[4]. According to Groh and co-authors, the presence of conduction disturbances, such as atrioventricular block (AVB) grade I and a clinical diagnosis of atrial tachyarrhythmia, are independent predictors of SCD [2]. Nevertheless, ECG, Holter and echocardiographic parameters are not sufficient for prediction of SCD.

Myocardial fibrosis has been identified in myocardial autopsies from patients with DM1 together with fat infiltration and myocyte hypertrophy and degeneration [5]-[7]. These findings are similar to observations in myocardial autopsies from other non-ischemic cardiomyopathies [5]-[8]. Myocardial fibrosis may not only explain the abnormalities in the cardiac conduction system but also work as a substrate for supraventricular and ventricular arrhythmias. Additionally, it may have a central role in the development of the systolic dysfunction observed in patients with DM1 [2],[7],[9],[10].

Cardiovascular magnetic resonance (CMR) is a well-established, non-invasive method to quantify heterogeneous myocardial fibrosis and assess left ventricular function and mass [11]. Screening procedures are needed for early detection of subclinical cardiac involvement in patients with DM1 and to identify those at high risk of SCD. CMR might become an essential screening-tool for identification of high-risk patients.

We investigated 30 unrelated and genetically verified DM1 patients with CMR and routine clinical screening to evaluate whether CMR add additional information about subclinical myocardial changes. In addition, our aim was to describe the prevalence and localization of myocardial fibrosis on CMR and to assess whether myocardial fibrosis was associated with abnormal findings on ECG, Holter-monitoring and echocardiography.

## Methods

### Study design

The study was conducted at the Departments of Cardiology and Neurology, Rigshospitalet, Copenhagen University Hospital, Rigshospitalet, Copenhagen, Denmark. We included a subgroup of patients (n = 30) from our cross-sectional study consisting of 129 genetically verified DM1 patients [4]. Except for CMR, methodology has previously been described in detail [4].

In brief, patients were evaluated with medical history, physical examination, 12-lead electrocardiogram (ECG), trans-thoracic echocardiography (including global longitudinal strain (GLS), 48-hour ECG-monitoring (Holter-monitoring) and CMR with late gadolinium enhancement (LGE). Blood samples were analyzed for plasma levels of NT-proBNP, myoglobin and creatine kinase (CK).

The study was approved by the regional scientific ethics committee (reference number H-d-2008-077) and all participating patients provided written informed consent.

### Study population

A total of 30 patients were included for CMR; 18 patients (10 men, mean age 51 years) with, and 12 patients (7 men, mean age 41 years) without abnormal findings on ECG and Holter-monitoring. Patients matched the main cohort in regard to age, gender and cardiac involvement and none of the patients were related. Patients with contraindications for CMR e.g. respiratory assist devices and claustrophobia were excluded, and few patients declined participation due to severe muscular impairment.

The diagnosis of DM1 (based on CTG-repeat length) was confirmed either by Southern blot analysis and/or by TP-PCR [1],[12].

### Cardiovascular magnetic resonance

**Figure 1:**
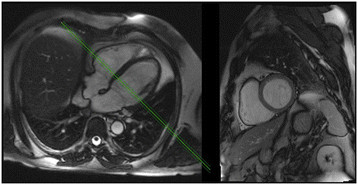
**CMR standard views.** CMR images with normal chamber dimensions (four-chamber view on the left and short-axis view on the right).

CMR was performed using a 1.5 Tesla magnetic resonance scanner using a 6-channel body array coil (Avanto, Siemens, Erlangen, Germany). Balanced steady-state free precession end-tidal breath hold cine images were acquired in the two-, three-, and four-chamber views followed by contiguous short-axis plane slices covering the entire left ventricle (LV) (echo time 1.5 ms, resolution matrix 192×192, field of view 300–360 mm, phases 25, slice thickness 8 mm without interpolated gap) (Figure 1). LV volume measurements were performed by tracing endocardial borders in the short-axis stack images. Correct time frames for LV end-diastolic volume (LVEDV) and LV end-systolic volume (LVESV) were automatically defined according to the size of the blood pool area, and LV ejection fraction (LVEF) was calculated accordingly. Papillary muscles were considered as part of the LV lumen. LV mass was measured at end-diastole by manually tracing the epicardial borders. To assess myocardial late gadolinium enhancement (LGE), we used T1-weightet inversion recovery gradient echo sequence (echo time 1.4 ms, resolution matrix 192×192 and field of view, 300–360 mm). Images were obtained 10 minutes after intravenous bolus injection of 0.1 mmol/kg body weight gadolinium-diethylenetriamine pentaacetic acid (Gadovist, Bayer Schering, Berlin, Germany). The inversion time was continuously determined to null the signal from normal myocardium. Multiple 8 mm slices in the short-axis image plane were acquired to cover the entire LV without gaps. Myocardial fibrosis of the left ventricle was defined as hyper-enhanced myocardium with a signal intensity (SI) above 5 standard deviations from the SI in the normal myocardium. The extent of hyper-enhancement in each slice is determined and added up for total extent in grams by the software cvi^42^ (Circle Cardiovascular Imaging Inc., Calgary, Canada). All analyses were performed by an experienced CMR physician (KA). Furthermore, all LGE images were concomitantly analyzed and results agreed upon by two experienced CMR physicians (KA and NV); both blinded for other findings. The inter-observer variation of LGE measurements has previously been described [13].

### Electrocardiography

A 12-lead ECG was performed using a Burdick Atria 6100 ECG, Richmond, Australia. An abnormal ECG was defined as the presence of; atrial flutter/fibrillation (AFL/AF), atrioventricular block (AVB) grade I–III (AVB grade I: PR-interval >220 ms), right and left bundle branch block (RBBB/LBBB), QRS-interval >120 ms, incomplete right bundle branch block (IRBBB) and prolonged QTc >450 ms in males and >470 ms in females using Bazett’s formula (QTc = QT/√RR).

### Holter-monitoring

A 48-hour Holter-monitoring was performed using a 3-electrode Lifecard CF (Spacelabs Healthcare, Washington, United States). Holter-monitoring was considered abnormal in the presence of; AVB grade I–III, AF/AFL, other supraventricular tachyarrhythmia (SVT) (>30 supraventricular premature contractions (SVPC) pr. hour or runs of ≥20 SVPC), frequent ventricular premature contractions (VPCs) (≥30/h) and non-sustained VT (NSVT) (minimum of 3 beats at ≥100 bpm).

### Transthoracic echocardiography and left ventricular longitudinal strain analysis

Transthoracic echocardiography was performed using a Vivid e9 (General Electric, Horten, Norway). LV cavity dimensions, mass and wall thickness and diastolic dysfunction were assessed in accordance with the recommendations of the European Association of Echocardiography and the American Society of Echocardiography [14],[15]. LV longitudinal function was assessed with global longitudinal strain (GLS) using a semiautomatic algorithm (Automated Function Imaging (AFI), GE), (normal reference GLS ≤ −15.9%) [16].

An abnormal echocardiography was defined as left ventricular ejection fraction ≤50%, left ventricular end diastolic diameter (LVEDD) >53 mm (women) and >59 mm (men), interventricular septum (IVS) >11 mm, left atrial volume indexed (LA vol.) >34 ml/m^2^ and GLS > −15.9%. Additionally, echocardiography was used to assess valve disease and to estimate LV mass [14].

### Muscle strength analysis

Muscle strength was graded using the Medical Research Council scale (MRC) 0–5 (0 = no ability to contract muscle, 5 = normal strength). Early affection occurs primarily in the distal muscles in patients with DM1. Therefore, we investigated the association between handgrip (dominant hand) and ankle dorsal flexion and the presence of myocardial fibrosis on CMR.

### Statistics

Data were analyzed with IBM SPSS Statistics version 19. Two sided p-values were calculated for all analyses; values ≤0.05 were considered statistically significant. Normally distributed values are expressed as means ± SD. Data with skewed distribution is given as median (range).

Categorical variables were summarized by frequency counts (percentage) and differences between groups were evaluated using chi-square test. Comparisons between categories were made with Mann–Whitney U test. Correlation analyses were performed using Spearman Correlation.

The patients included for CMR (n=30) were divided into two groups: one with (n=18) and one without (n=12) abnormal findings on ECG and Holter-monitoring to evaluate whether abnormal findings on conventional cardiac assessment were associated with fibrosis. Sample size calculation was based on the hypothesis that a minority of patients without abnormal findings on ECG and Holter-monitoring would have myocardial fibrosis (maximum prevalence 5%). In contrast, we would expect myocardial fibrosis to be present in the majority of patients (app. prevalence 60%) with abnormal findings on ECG and Holter-monitoring, corresponding to a total sample size of 28 patients with a power of 90%, and a two-sided alpha-level of 5%.

## Results

### Study population

A total of 30 patients (17 men with a mean (SD) age of 47 (14) years) were included for CMR: 18 patients with, and 12 patients without abnormal findings on ECG and Holter-monitoring (Table 1). Patients with abnormal findings on ECG and/or Holter had higher NT-proBNP (Table 1). None complained of cardiac symptoms including palpitations, dizziness, chest pain, dyspnoea, peripheral oedema or syncope.

**Table 1:** Clinical and cardiac findings in patients with DM1 prior to CMR

**Demographics**	**Patients without abnormal findings on ECG/Holter**	**Patients with abnormal findings on ECG/Holter**	**p-value**
N	12	18	-
Gender	7 men, 5 women	10 men, 8 women	1.00
Age (years), mean (SD)	41 (10)	51 (15)	0.05
**Vital signs**			
SBP (mmHg), mean (SD)	115 (11)	126 (19)	0.09
DBP (mmHg), mean (SD)	74 (10)	76 (11)	0.64
**Cardiac medications**			
B-blocker (n)	1	1	1.00
ACEI (n)	0	3	0.24
Beta-blocker + ACEI (n)	0	1	1.00
**Laboratory Data**			
NT-ProBNP (pmol/l), median (range)	8 (1–29)	13 (1–41)	0.02
Myoblobin (ug/l), median (range)	68 (27–255)	103 (44–258)	0.22
CK (U/l), median (range)	171 (42–498)	182 (71–780)	0.43
**Muscle strength testing**			
Handgrip (MRC), median (range)	4.3 (2–5)	4.0 (1–5)	0.59
Ankle dorsal flexion (MRC), median (range)	5.0 (2–5)	4.5 (1–5)	0.45
**ECG**			
PR interval (ms), median (range)	179 (130–200)	240 (160–260)	0.006
QRS interval (ms), median (range)	88 (70–110)	96 (78–160)	0.24
QTc interval (ms), median (range)	394 (346–451)	417 (360–516)	0.06
AVB grade I (n)	0	10	0.002
LBBB (n)	0	1	1.00
IRBBB (n)	0	4	0.13
**Holter-monitoring**			
AVB grade II (n)	0	3	0.25
AF/AFL (n)	0	4	0.13
SVT (n)	0	2	0.50
NSVT (n)	0	1	1.00
VPC/h, median (range)	0 (0–15)	4 (0–421)	0.004
**Echocardiography**			
LVEF (%), median (range)	57 (48–67)	58 (45–67)	0.72
IVS (mm), median (range)	8 (6–13)	8 (5–11)	0.87
LVEDD (mm), median (range)	46 (40–60)	47 (41–56)	0.97
LVPW (mm), median (range)	7 (6–12)	8 (5–11)	0.70
LV mass (g/m^2^), median (range)	70 (49–90)	70 (46–141)	0.90
LA vol. (ml/m^2^), median (range)	22 (14–36)	25 (17–38)	0.50
GLS avg. (%), median (range)	−19 (−14 to −23)	−18 (−14 to −25)	0.66

ACEI: angiotensin converting enzyme inhibitor.

AF/AFL: atrial fibrillation/flutter.

AVB: atrioventricular block.

B-blocker: beta-blocker.

CK: creatine kinase.

DBP: diastolic blood pressure.

GLS: global longitudinal strain.

IRBBB: incomplete right bundle branch block.

IVS: interventricular septum.

LA vol.: left atrial volume.

LBBB: left bundle branch block.

LVEDD: left ventricular end diastolic diameter in diastole.

LVEF: left ventricular ejection fraction.

LV mass: left ventricular mass.

LVPW: left ventricular posterior wall.

MRC: medical research council scale.

NSVT: non-sustained ventricular tachycardia.

SVT: supraventricular tachycardia.

SBP: systolic blood pressure

VPC/h: ventricular premature contractions/hour.

### Cardiovascular magnetic resonance

**Figure 2:**
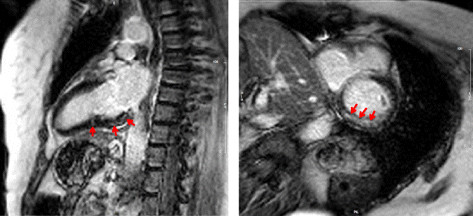
**Late gadolinium enhancement.** Representative images (long-axis on the left and short-axis on the right) of late gadolinium enhancement (marked with red arrows) in a 63-year-old woman with concomitant atrioventricular block grade I and frequent ventricular premature contractions on Holter-monitoring.

**Figure 3:**
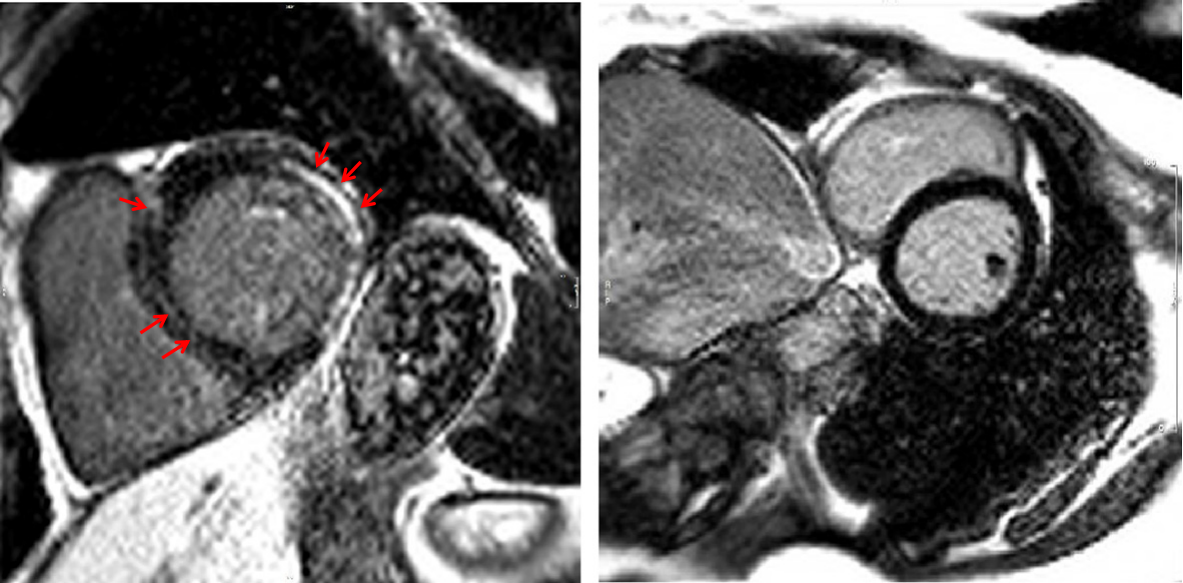
**Late gadolinium enhancement.** Representative short-axis image (left) showing late gadolinium enhancement of the lateral and posterior left ventricular wall, and of the midwall and posterior hinge point (red arrows) in a 58-year-old man with concomitant AVB grade I. In comparison, normal findings are illustrated on the right.

Myocardial fibrosis of the left ventricle was found in 12/30 patients (40%, 9 men) with a median LGE quantity of 4 g (range 1–17 g) (Figures 2 and 3). All patients were in sinus rhythm at time of the CMR. Gender specific CMR results are presented in Table 2.

**Table 2:** Gender-specific CMR results in patients with DM1

	**All**	**Men**	**Women**
Myocardial fibrosis, n (%)	12 (40)	9 (75)	3 (25)
LGE (g), median (range)	4 (1–17)	4 (3–17)	3 (1–10)
LVEF (%), median (range)	63 (38–71)	60 (38–65)	66 (63–71)
LVEF ≤ 50%, n (%)	2 (17)	2 (22)	0
LVEDV (ml/m^2)^, median (range)	83 (53–130)	89 (61–130)	75 (53–75)
LVESV (ml/m^2^), median (range)	31 (15–73)	33 (22–73)	26 (15–28)
LV mass (g/m^2^), median (range)	55 (43–83)	58 (47–83)	44 (43–4550)
LA vol. (ml/m^2^), median (range)	52 (36–87)	56 (46–87)	45 (36–49)

LGE: late gadolinium enhancement.

LV mass: left ventricular mass.

LVEDD: left ventricular end diastolic diameter in diastole.

LVEDV: left ventricular end diastolic volume.

LVEF: left ventricular ejection fraction.

LVESV: left ventricular end systolic volume.

The presence of myocardial fibrosis, independently of quantity, was associated with the following CMR parameters: increased left ventricular mass, increased left atrial volume and a trend toward lower LVEF (Table 3). Two patients had reduced LVEF of 38% and 50% and concomitant myocardial fibrosis. Myocardial fibrosis did not correlate with age (Spearman correlation r = 0.20, p = 0.28).

**Table 3:** Clinical characteristics and CMR-results in patients with and without cardiac fibrosis

**Clinical characteristics**	**Fibrosis (n = 12)**	**No fibrosis (n = 18)**	**p-value**
Age (years), mean (SD)	51 (18)	44 (10)	0.28
SBP (mmHg), mean (SD)	128 (104–168)	117 (97–135)	0.17
DBP (mmHg), mean (SD)	78 (65–95)	73 (56–101)	0.14
NT-ProBNP (pmol/l), median (range)	9 (1–18)	11 (1–41)	0.38
Myoblobin (ug/l), median (range)	103 (34–162)	69 (27–258)	0.56
CK (U/l), median (range)	199 (56–498)	162 (42–780)	0.61
Handgrip (MRC), median (range)	3.8 (1–5)	4.0 (2–5)	0.68
Ankle dorsal flexion (MRC), median (range)	5 (1–5)	4.5 (3–5)	0.77
**CMR-results**			
LVEF%, median (range)	63 (38–71)	66 (60–80)	0.06
LGE (g), median (range)	4 (1–17)	0	<0.001
LVEF ≤ 50%, n (%)	2 (17)	0	0.15
LVEDV (ml/m^2^), median (range)	83 (53–130)	71 (57–108)	0.16
LVESV (ml/m^2^), median (range)	31 (15–73)	25 (13–43)	0.03
LV mass (g/m^2^), median (range)	55 (43–83)	46 (36–64)	0.02
LA vol. (ml/m^2^), median (range)	52 (36–87)	46 (35–69)	0.04
**ECG**			
PR interval (ms), median (range)	191 (160–220)	190 (130–260)	0.85
QRS interval (ms), median (range)	96 (70–160)	86 (78–120)	0.43
QTc interval (ms), median (range)	411 (360–516)	407 (346–478)	0.66
AVB grade I	3	7	0.69
LBBB	1	0	0.40
IRBBB	4	0	0.02
**Holter-monitoring**			
AVB grade II	0	3	0.25
AF/AFL	3	1	0.27
SVT	2	0	0.15
NSVT	1	0	0.40
VPC/h, median (range)	3 (0–421)	0 (0–69)	0.30
**Echocardiography**			
LVEF (%), median (range)	57 (45–53)	58 (48–67)	0.34
IVS (mm), median (range)	8 (5–11)	8 (6–13)	0.83
LVEDD (mm), median (range)	50 (40–60)	46 (40–60)	0.24
LVPW (mm), median (range)	8 (5–11)	7 (6–12)	0.77
LV mass (g/m2), median (range)	74 (47–141)	68 (46–87)	0.74
LA vol. (ml/m^2^), median (range)	26 (17–38)	24 (14–29)	0.08
GLS avg. (%), median (range)	−19 (−14 to −23)	−18 (−14 to −25)	0.75
E/A	1.2 (0.6-2.6)	1.4 (0.8-2.4)	0.69
E/E’ (lateral)	5.4 (3.5-7.4)	4.9 (3.6-8.2)	0.39
MV dec. time (ms)	150 (91–214)	157 (107–239)	0.81

ACEI: angiotensin converting enzyme inhibitor.

AF/AFL: atrial fibrillation/flutter.

AVB: atrioventricular block.

B-blocker: beta-blocker.

CK: creatine kinase.

DBP: diastolic blood pressure.

GLS: global longitudinal strain.

IRBBB: incomplete right bundle branch block.

IVS: interventricular septum.

LA vol: left atrial volume.

LBBB: left bundle branch block.

LGE: late gadolinium enhancement.

LV mass: left ventricular mass.

LVEDD: left ventricular end diastolic diameter in diastole.

LVEDV: left ventricular end diastolic volume.

LVEF: left ventricular ejection fraction.

LVESV: left ventricular end systolic volume.

LVPW: left ventricular posterior wall.

MRC: medical research council scale.

MV dec. time: mitral valve deceleration time.

NSVT: non-sustained ventricular tachycardia.

SBP: systolic blood pressure.

SVT: supraventricular tachycardia.

VPC/h: ventricular premature contractions/hour.

Myocardial fibrosis was heterogeneously located in the left ventricle: anterior (n = 2), posterior (n = 1), anterior-septal (n = 3), posterior-basal (n = 1) and lateral segments (n = 2). Of these nine patients, six had concomitant fibrosis of the anterior (n = 1), posterior (n = 4) and both hinge points (n = 1) between the right and left ventricle. Three patients had isolated prominent hinge point fibrosis, related to the anterior, posterior, and both hinge points, respectively. No fibrotic lesions were observed in the right ventricle.

### ECG

With regard to the pre-selected DM1 subgroups, myocardial fibrosis was found in 8/18 (44%) of the patients with abnormal findings on ECG and Holter-monitoring and interestingly in 4/12 (33%) of the patients with normal findings on ECG and Holter (p = 0.71).

Selected ECG, echocardiographic and Holter-monitoring results in patients with and without myocardial fibrosis are summarized in Table 3. Myocardial fibrosis was associated with IRBBB. There was no association between the presence of myocardial fibrosis and the following ECG parameters: AVB grade I, LBBB and prolonged QTc (Table 3). Overall, 7/30 patients (23%) had myocardial fibrosis and one or several abnormal findings on the ECG vs. 5/30 (17%) patients with myocardial fibrosis and normal ECG (p = 0.71).

The quantity of LGE did not correlate with the following parameters: PR interval (Spearman correlation r = 0.01, p = 0.97), QRS interval (r = 0.23, p = 0.22) or QTc-interval (r = 0.10, p = 0.59).

### Holter-monitoring

The prevalence of abnormal findings on Holter-monitoring in patients with (n = 12) and without fibrosis (n = 18) was: AF/AFL (3/12 vs. 1/18, p = 0.27), SVT (2/12 vs. 0/18, p = 0.15), AVB grade II (0/12 vs. 3/18, p = 0.25), frequent VPC (2/12 vs. 2/18, p = 1.00), NSVT (1/12 vs. 0/18, p = 0.40). As reported, three patients (men, aged 46, 53 and 68 years) had paroxysmal atrial fibrillation and concomitant myocardial fibrosis, and one patient (a 46-year-old man) had permanent atrial fibrillation and flutter, which was terminated with radiofrequency ablation prior to CMR. Overall, no association was observed between myocardial fibrosis and abnormal findings on Holter-monitoring, i.e. 6/30 (20%) had myocardial fibrosis and abnormal Holter-monitoring vs. 6/30 (20%) with myocardial fibrosis and normal Holter-findings (p = 0.27).

The quantity of LGE did not correlate with the number of VPC/h (Spearman correlation r = 0.24, p = 0.20).

### Echocardiography

When we added echocardiographic findings to the pre-selected DM1-subgroups, a total of 21/30 patients had abnormal findings on ECG, Holter-monitoring and/or echocardiography, and of these 21 patients, myocardial fibrosis was present in 9 (43%). Of the remaining 9 patients with normal routine cardiac screening 3 (33%) had myocardial fibrosis. Taken together, no statistically significant association was observed between the presence of myocardial fibrosis and cardiac involvement on routine cardiac screening (p = 0.70).

There was no overall association between abnormal findings on echocardiography and myocardial fibrosis on CMR, i.e. 4/30 (13%) had myocardial fibrosis and abnormal echocardiography vs. 8/30 (27%) with myocardial fibrosis and normal echocardiography (p = 0.18). Furthermore, there was no association between myocardial fibrosis and the following specific echocardiographic parameters: LVEF ≤50% (2/12 vs. 1/18, p = 0.55), IVSD >11 mm (1/12 vs. 1/18, p = 1.00), abnormal LVEDD (3/12 vs. 2/18, p = 0.36), LA vol. >34 ml/m^2^ (2/10 vs. 0/18, p = 0.12) and abnormal GLS (3/10 vs. 2/16, p = 0.34).

The quantity of myocardial fibrosis correlated significantly with LA volume (Spearman correlation r = 0.40, p = 0.03), but there was no correlation with the remaining echocardiographic parameters: IVSD (r = −0.10, p = 0.59), LVIDD (r = 0.25, p = 0.18), LVEF (r = −0.19, p = 0.31), E/E’ (r = 0.14, p = 0.50), E/A (r = −0.05, p = 0.79), GLS (r = 0.13, p = 0.54) and LV mass (r = 0.05, p = 0.81). One patient (a 57-year-old man) had increased LV mass of 141 g/m^2^ and concomitant myocardial fibrosis.

Two patients with fibrosis had increased LA volume (36 and 38 ml/m^2^, respectively) but no other signs of diastolic dysfunction. Echocardiography excluded significant valve disease.

## Discussion

This study demonstrates that adult patients with DM1 have a high prevalence of myocardial fibrosis (40%). Myocardial fibrosis was associated with increased left ventricular mass, increased LA volume and a trend toward lower LVEF assessed by CMR. On standard cardiac screening, myocardial fibrosis was associated with IRBBB and correlated with LA volume assessed by echocardiography. Nevertheless, a normal ECG, echocardio-graphy and/or Holter-monitoring could not rule out the presence of myocardial fibrosis on CMR.

Physicians treating and referring patients with DM1 face a major challenge in handling the risk of SCD in these patients. Risk predictors for SCD in patients with DM1 are needed and specific ECG abnormalities, such as atrial tachyarrhythmia and AVB, are already known predictors of SCD [2]. Studies have reported a correlation between QRS-interval and myocardial fibrosis and an overall association between ECG abnormalities and abnormal CMR findings in DM1 patients [17]-[19]. Nevertheless, myocardial fibrosis in DM1 patients has only been investigated in few studies [17]-[21]. Although these studies are not directly comparable due to different CMR-techniques, myocardial fibrosis has been reported with prevalence ranging from 10 to 40%, i.e. in the order of magnitude as presently found. Our study is the first to evaluate the association between myocardial fibrosis on CMR and a systematic standard cardiac screening including ECG, Holter-monitoring and echocardiography. A recent CMR-study including 80 patients with DM1 reported myocardial fibrosis in 10 patients (13%) and 9/10 had concomitant abnormal ECG [18]. In comparison, we found a higher prevalence of patients with myocardial fibrosis (40%) and no association between myocardial fibrosis and abnormal ECG findings. The above findings substantiate that myocardial fibrosis has an impact on the pathogenic process of DM1, although not necessarily related to the findings based on routine cardiac evaluation [17]-[21].

In several cardiomyopathies, myocardial fibrosis seems to act as a substrate for ventricular arrhythmias. Furthermore, myocardial fibrosis is a dominant finding in endomyocardial biopsies from patients with spontaneous ventricular fibrillation and no other macroscopic cardiac disease [22]-[26]. Additionally, studies have demonstrated an association between the quantity of myocardial fibrosis and the risk of ventricular arrhythmias [27]-[30]. In patients with DM1, myocardial fibrosis has been observed in autopsy findings and in pathology studies with animal-models of DM1 [6]-[8],[31]-[35], giving evidence of a possible link between myocardial fibrosis and ventricular arrhythmias, which may lead to SCD. Skeletal muscle biopsies have also revealed fibrotic changes together with muscle fiber diameter variation, adipose deposition and a high number of central nuclei [36]. So far, there is no definitive pathogenic explanation for these histopathological alterations, although combined effects of mis-regulated splicing of several genes involved in calcium regulation and extracellular coupling may contribute to the muscle degeneration [37],[38]. Nonetheless, myocardial fibrosis seems to have a central role in the pathogenic mechanism in both skeletal and cardiac muscle. In patients with hypertrophic cardiomyopathy, hypertrophy and interstitial fibrosis are important determinants of morbidity and mortality [39],[40] and the changes are suggested to occur as a response to trophic and fibrotic factors such as levels of angiotensin II [41]. Transforming growth factor (TGF)-β is an important mediator of pro-fibrotic signals, and early inhibition of TGF-β e.g. with ACE-inhibitors has shown to diminish the development of fibrosis and hypertrophy independent of blood pressure [42]. A recent case-report presented a 50-year-old DM1 patient who suddenly died due to malignant arrhythmia [35]. Autopsy revealed a normal sized heart with no atherosclerotic lesions, but patchy fibrosis involving 20% of the left and right ventricular myocardium and a strong immune-positive result of TGF-β expression. This case was considered by the authors to indicate an association between TGF-β and the fibrotic lesions in DM1. In addition, studies have shown that ACE-inhibitors, or ACE-inhibitors in combination with beta-blockers, delay the progression of cardiomyopathy in patients with Duchenne muscular dystrophy (DMD), and has a beneficial effect on long-term survival of DMD patients with heart failure [40],[43]. As there are some phenotypic similarities between patients with DM1 and DMD, a similar beneficial pharmacological effect may exist in patients with DM1. Early treatment with ACE-inhibitors should therefore be considered and need further investigation in relation to SCD in DM1.

So far, no homogeneous LGE pattern has been described in DM1, which is in accordance with our findings [18],[20]. However, it seems that mid-myocardial enhancement is most often observed in the septal and basal lateral segments [18],[20]. Mid-wall fibrosis in patients with non-ischaemic dilated cardiomyopathy provides independent prognostic information and improves risk stratification beyond LVEF for all-cause mortality and SCD [44]. Isolated RV-insertion point enhancement was observed in three patients, which can also be observed in healthy individuals, usually as tiny faint enhancement. However, the insertion point fibrosis observed in these patients was prominent and more pronounced than what is seen in healthy individuals. We need to reassess our DM1 patients with CMR, before we can document the prognostic information related not only to the localization but also to the progression of myocardial fibrosis in DM1.

Taken together, myocardial fibrosis may play a central role in the pathogenic process in DM1. Our findings emphasize that CMR add additional information to current screening procedures with a potential major impact on future risk stratification for SCD. Assuming that myocardial fibrosis is a predictor of cardiac outcome, CMR may be a unique tool for identifying high-risk DM1 patients in order to optimize early treatment and prevent SCD.

Longitudinal studies are needed to investigate the prognostic value of myocardial fibrosis and to assess whether improved periodic cardiac assessment, based on the presence, progression and degree of myocardial fibrosis, can prevent life-threatening brady- and tachyarrhythmias through optimized medical treatment and implantation of PMs and ICDs.

### Study limitations

Contrast enhanced CMR is a well-established technique to assess focal myocardial fibrosis. We did not assess the degree of diffuse myocardial fibrosis, hence, we may underestimate and even oversee the fibrotic burden in some patients.

Some patients were not eligible for CMR due to ventilatory assist devices or claustrophobia and few patients refused to participate due to severe muscular impairment. The exclusion of patients with the most severe muscular and respiratory impairment represents selection bias. However, we believe this would be a common bias for all CMR studies in this field. Furthermore, if these patients would have more cardiac involvement than the remaining patients, it would probably skew our results towards substantiating our conclusion that myocardial fibrosis is a frequent finding in patients with DM1.

Several studies have assessed the correlation between CTG repeat length and cardiac involvement with ambiguous results [3],[45]. A recent study reported a large inter- and intra-tissue CTG length variation in DM1 tissue with the largest expansion in the heart and cerebral cortex [46]. These observations strongly indicate that factors other than genetic are responsible for the observed cardiac involvement in DM1 and explain why CTG-repeat length is only estimated for diagnostic purposes.

Due to the relatively small number of patients in our study, the impact of our findings on the clinical management of patients with DM1 is limited and needs to be assessed in larger studies.

## Conclusion

CMR documented a high prevalence of myocardial fibrosis in patients with DM1. Overall, myocardial fibrosis was mainly observed in patients with concomitant abnormal findings on ECG, Holter-monitoring and/or echocardio-graphy. Nevertheless, myocardial fibrosis was also present in 33% of the patients with normal findings on routine cardiac screening. These findings emphasize that a normal ECG, Holter-monitoring and echocardiography cannot exclude myocardial fibrosis.

CMR adds additional information to the current standard cardiac assessment and might be a valuable tool for risk stratification in DM1. The progression and the prognostic value of myocardial fibrosis needs to be further investigated in longitudinal studies.

## Competing interests

The authors declare that they have no competing interests.

## Authors’ contributions

HP, HB and LK were responsible for the study design. HP and KAA collected data. HP, JV, HB, KAA and LK contributed to data analysis. All authors contributed to data interpretation and preparation of the manuscript. HP and HB are responsible for the overall content of the manuscript. All authors read and approved the final manuscript.
